# Impact of V179D/E mutations on antiretroviral therapy outcomes in people living with HIV-1: a 3-year retrospective study

**DOI:** 10.3389/fcimb.2025.1691715

**Published:** 2025-12-04

**Authors:** Shiyun Lv, Yun Lan, Quanmin Li, Xuemei Ling, Junbin Li, Chunyan Wen, Yonghong Li, Jingliang Chen, Xiejie Chen, Weiping Cai, Xiaoping Tang, Linghua Li

**Affiliations:** 1 Infectious Disease Center, Guangzhou Eighth People’s Hospital, Guangzhou Medical University, Guangzhou, Guangdong, China; 2 Guangzhou Medical Research Institute of Infectious Diseases, Guangzhou Eighth People’s Hospital, Guangzhou Medical University, Guangzhou, Guangdong, China; 3 Institute of Infectious Disease, Guangzhou Eighth People’s Hospital, Guangzhou Medical University, Guangzhou, Guangdong, China; 4 Guangzhou Key Laboratory of Clinical Pathogen Research for Infectious Diseases, Guangzhou Medical University, Guangzhou, Guangdong, China

**Keywords:** HIV-1, pre-treatment drug resistance, mutation, V179D, V179E, efficacy

## Abstract

**Background:**

HIV-1 mutation V179D/E can confer potential low-level resistance to multiple non-nucleoside reverse transcriptase inhibitors (NNRTIs), and its detection rate has increased in recent years. However, its effect on antiretroviral therapy (ART) outcomes remains unclear.

**Methods:**

This study included people living with HIV-1 (PLWH) with only V179D/E mutation detected by pre-treatment drug resistance (PDR) testing at Guangzhou Eight People’s Hospital between January 2018 and December 2022. Two control groups were matched 1:1:1 using propensity score matching (PSM): a PDR-negative group and an NNRTI-DR group with low-level or higher NNRTI resistance. Virological and immunological outcomes were compared over 3 years. Logistic regression analyzed virological failure (VF) risk factors in the V179D/E group and assessed acquired drug resistance (ADR).

**Results:**

Among 6021 patients tested, the detection rate of V179D/E was 14.8%. After exclusions, 626 patients were included in this study. Additionally, 195 patients met the criteria for the NNRTI-DR group. After 1:1:1 PSM, the baseline characteristics were balanced across the three groups. In 1 year, the V179D/E group showed lower virological suppression and higher VF than the PDR-negative group, with no significant difference from the NNRTI-DR group. Differences disappeared by years 2 and 3. In the V179D/E group, NNRTI-based regimens increased VF risk, while baseline CD4+ T cell counts >200 cells/μL were protective. Among 37 patients with VF tested for ADR in the V179D/E group, 54.1% developed new mutations, 85.0% of whom were on efavirenz (EFV)-based regimens.

**Conclusions:**

V179D/E is highly prevalent among ART-naïve PLWH in Guangzhou and may impair early virological response to NNRTI-based regimens, particularly EFV-based regimens while increasing ADR risk.

## 1 Introduction

HIV-1 exhibits a remarkably high mutation rate and extensive genetic diversity, which facilitates the emergence of drug-resistant variants and poses a significant threat to the long-term efficacy of antiretroviral therapy (ART). According to the World Health Organization (WHO) guidelines (World Health Organization, 2019), HIV drug resistance is classified into acquired drug resistance (ADR), transmitted drug resistance (TDR), and pre-treatment drug resistance (PDR). PDR refers to the presence of resistance-associated mutations detected at the initiation or re-initiation of ART in individuals with no prior ART exposure or only limited exposure to antiretroviral (ARV) drugs.

Among the various classes of ARVs, non-nucleoside reverse transcriptase inhibitors (NNRTIs) are particularly prone to the development of drug resistance because of their low genetic barrier (Steegen et al., 2017; Taffa et al., 2018). Globally, the prevalence of NNRTI-associated PDR has increased significantly since 2001, with several countries reporting rates exceeding 10% among individuals initiating ART (World Health Organization, 2019). Despite this trend, NNRTIs remain widely used in first-line treatment regimens (Panel on Antiretroviral Guidelines for Adults and Adolescents, 2024; European AIDS Clinical Society Guidelines, 2024), underscoring the importance of continued surveillance of NNRTI-associated resistance.

HIV drug resistance is commonly evaluated using the Stanford HIV Drug Resistance Database algorithm in clinical practice (HIVDB Genotypic Resistance Test Interpretation System, 2019), which categorizes resistance into five levels: susceptible, potential low-level resistance, low-level resistance, intermediate-level resistance, and high-level resistance. Among these, low-level resistance and higher are generally considered clinically significant and may lead to virological failure (VF) (Jespersen et al., 2018), whereas the clinical impact of potential low-level resistance remains unclear.

V179D and V179E are two accessory mutations associated with NNRTI resistance, both of which can confer potential low-level resistance to multiple NNRTIs. V179D is a polymorphic mutation that can act synergistically with the K103R mutation, significantly enhancing resistance to efavirenz (EFV) and nevirapine (NVP). V179E is a non-polymorphic mutation, although both exhibit similar resistance profiles under drug pressure. In recent years, the prevalence of V179D/E has continued to increase (Li et al., 2016). In our previous study, we found that the overall prevalence of PDR in Guangzhou, China, was 7.4%, with NNRTI resistance being the most common. Among these, V179D/E mutation was the most frequently detected, with a prevalence of 17.8% (Lv et al., 2024).

Preliminary studies have suggested that people living with HIV-1 (PLWH) harboring V179D/E may exhibit poorer virological suppression (VS) and immune reconstitution when treated with EFV-based regimens, whereas regimens based on protease inhibitors (PIs) or integrase strand transfer inhibitors (INSTIs) tend to yield better outcomes (Wang et al., 2023). However, evidence regarding the long-term clinical impact of V179D/E remains limited. Therefore, this study aimed to assess the prevalence of V179D/E among ART-naïve patients with HIV-1 in Guangzhou, China, and to investigate their influence on antiretroviral treatment outcomes, with the goal of informing the optimization of initial ART regimens and resistance management strategies.

## 2 Methods

### 2.1 Study design and participants

This study was a retrospective cohort study. The study population consisted of PLWH who underwent PDR testing at Guangzhou Eighth People’s Hospital, Guangzhou Medical University (GZ8H), between January 2018 and December 2021, and were found to carry only a single V179D or V179E mutation. Inclusion criteria were as follows: 1) confirmed diagnosis of HIV-1; 2) detection of V179D or V179E as the only mutation in PDR, with no other drug resistance mutations (DRMs) identified; and 3) initiation and continuous receipt of ART at GZ8H, with complete follow-up data for a minimum of 3 years. Patients were excluded if they were lost to follow-up, died, discontinued ART, or were transferred to other medical institutions during the follow-up period.

To assess the impact of V179D/E on virological and immunological outcomes over a 3-year follow-up period, two control groups were selected from the same study period. The PDR-negative group included patients in whom no DRMs were identified in PDR, while the NNRTI-DR group included patients who were found to exhibit low-level resistance or higher to NNRTIs only, with full susceptibility to all other classes of ARVs. The three groups were matched in a 1:1:1 ratio using propensity score matching (PSM). Matching variables included sex, age, baseline CD4^+^ T cell count, and initial ART regimen to minimize baseline differences. Baseline viral load (VL) was not included due to a high rate of missing data.

### 2.2 Data collection and definitions

Demographic and clinical data for the three study groups were obtained from the National Free Antiretroviral Treatment Database for Disease Control and Prevention. The collected baseline information included age, sex, route of HIV transmission, WHO clinical stage, baseline CD4^+^ T cell count, baseline plasma VL, and initial ART regimen. Additionally, CD4^+^ T cell counts and VL measurements were collected annually during the three-year follow-up period for all three groups. For patients in the V179D/E group, any changes in ART regimen during the follow-up period were also recorded.

VS was defined as a plasma VL of <50 copies/mL after at least 24 weeks of ART. VF was defined as plasma VL persistently >200 copies/mL 6 months after the initiation or adjustment of treatment, or virological rebound, defined as the recurrence of VL >200 copies/mL after full VS has been achieved (Acquired Immunodeficiency Syndrome Professional Group et al., 2024).

### 2.3 Analysis of HIV-1 subtype and drug resistance

Plasma samples were subjected to RNA extraction and reverse transcription, followed by nested polymerase chain reaction (PCR) amplification of the HIV-1 *pol* gene fragment, which includes the entire protease region and the first 240 amino acids of the reverse transcriptase region (corresponding to HXB2 positions 2253 to 3318), as previously described (Lv et al., 2024). The PCR products were subsequently analyzed using Sanger sequencing. Sequence data were assembled and edited using Sequencer version 5.0 and manually corrected with BioEdit version 7.2.6.1. HIV-1 subtypes were determined using the HIV-1 BLAST and the COMET online platform. Sequences were submitted to the Stanford HIV Drug Resistance Database to identify DRMs and assess ARV susceptibilities. The degree of drug resistance was classified into five categories: susceptible, potential low-level, low-level, intermediate-level, and high-level. Drug resistance was defined as low-level or higher in this study.

### 2.4 Statistical analysis

Continuous variables were presented as medians with interquartile range (IQR), while categorical variables were summarized as frequencies and percentages. Group comparisons of categorical variables were conducted using the Pearson chi-square test, and continuous variables were analyzed using the Wilcoxon rank-sum test for nonparametric analysis. To identify risk factors associated with VF in the V179D/E group, univariable and multivariable logistic regression models were constructed, and odds ratio (OR), adjusted OR (aOR), and corresponding 95% confidence interval (CI) were reported. All statistical tests were two-sided, and a *P*-value of <0.05 was considered statistically significant. PSM was performed using R software (version 4.4.1, R Core Team, Vienna, Austria). All other statistical analyses were conducted using SPSS software (IBM SPSS Statistics, version 26.0).

### 2.5 Ethical statement

This study was approved by the ethics committee of Guangzhou Eighth People’s Hospital, Guangzhou Medical University (GZ8H) (approval number: 202033166). Each participant signed a written consent form.

## 3 Results

### 3.1 Prevalence of V179D/E mutations and baseline characteristics of carriers

In total, 6021 patients underwent PDR testing during the study period. Among them, 14.8% (893/6021) were found to carry either V179D or V179E. Specifically, the detection rate of V179D was 4.3% (259/6021), while that of V179E was 10.5% (634/6021). After excluding patients who had discontinued ART, were lost to follow-up, or were referred elsewhere, 626 patients were included in the final analysis.

The median age of the included patients was 40.0 years (IQR: 31.0–52.0), with males accounting for 93.6% (586/626). The predominant route of transmission was same-sex sexual behavior, accounting for 63.4% (397/626) of the cohort. Most patients (90.1%, 564/626) were classified as WHO clinical stage III or IV. The median baseline CD4^+^ T cell count was 208.0 cells/μL (IQR: 70.8–325.3), and the median baseline VL was 5.2 log10 copies/mL (IQR: 4.7–5.8). At baseline, 63.6% (398/626) of the patients received an ART regimen consisting of two nucleoside reverse transcriptase inhibitors (NRTIs) plus an NNRTI, of which 99.5% (396/398) received an EFV-based regimen. Notably, 85.4% (386/452) of those with the V179E mutation harbored CRF55_01B. For specific details, refer to **Table 1**. 

**Table 1:** Baseline characteristics of patients with V179D/E.

Characteristic	Total (n=626)	V179D (n=174)	V179E (n=452)
Age, median (IQR)	40.0 (31.0-52.0)	38.0 (32.0-53.0)	41.0 (31.0-52.0)
Sex			
Male	586 (93.6%)	155 (89.1%)	431 (95.4%)
Female	40 (6.4%)	19 (10.9%)	21 (4.6%)
Route of infection			
same-sex sexual behavior	397 (63.4%)	100 (57.5%)	297 (65.7%)
Heterosexual intercourse	185 (29.6%)	61 (35.1%)	124 (27.4%)
Others	44 (7.0%)	13 (7.5%)	32 (7.1%)
WHO clinical stage			
I and II	62 (9.9%)	18 (10.3%)	44 (9.7%)
III and IV	564 (90.1%)	156 (89.7%)	408 (90.3%)
CD4^+^ T cell count before ART (cells/μL), median (IQR)	208.0 (70.8-325.3)	146.0 (34.0-309.3)	221.0 (100.3-338.8)
VL before ART (log10 copies/mL), median (IQR)^a^	5.2 (4.7-5.8)	5.1 (4.6-5.7)	5.3 (4.7-5.8)
ART regimen at baseline			
2NRTIs+NNRTI^b^	398 (63.6%)	101 (58.0%)	297 (65.7%)
2NRTIs+PI	51 (8.1%)	17 (9.8%)	34 (7.5%)
2NRTIs+INSTI	155 (24.8%)	49 (28.2%)	106 (23.5%)
Simplified regimen	22 (3.5%)	7 (4.0%)	15 (3.3%)
Subtype			
CRF01_AE	104 (16.6%)	77 (44.3%)	27 (6.0%)
CRF07_BC	82 (13.1%)	62 (35.6%)	20 (4.4%)
CRF55_01B	396 (63.3%)	10 (5.7%)	386 (85.4%)
B	10 (1.6%)	8 (4.6%)	2 (0.4%)
CRF08_BC	12 (1.9%)	10 (5.7%)	2 (0.4%)
Others	22 (3.5%)	7 (4.0%)	15 (3.3%)

IQR, interquartile range; WHO, World Health Organization; ART, antiretroviral therapy; VL, viral load; NRTI, nucleoside reverse transcriptase inhibitor; NNRTI, non-nucleoside reverse transcriptase inhibitor; PI, protease inhibitor; INSTI, integrase strand transfer inhibitor; CRF, circulating recombinant form; 3TC, lamivudine; TDF, tenofovir disoproxil fumarate; EFV, efavirenz; ABC, abacavir; AZT, zidovudine; NVP, nevirapine.

^a^The V179D and V179E groups include 71 and 191 individuals, respectively; ^b^Among the 398 patients receiving 2NRTI + NNRTI regimens, 384 patients received 3TC + TDF + EFV, 3 patients received EFV + 3TC + ABC, 9 patients received EFV + AZT/3TC, and 2 patients received 3TC + TDF + NVP.

### 3.2 Comparison of virologic and immunologic responses among the V179D/E, PDR-negative, and NNRTI-DR groups, including V179D and V179E subgroup analysis

During the study period, 275 patients met the inclusion criteria for the NNRTI-DR group. After excluding 80 patients due to loss to follow-up, ART interruption, or referral, 195 patients were included. The specific NNRTI-associated mutations and corresponding resistance levels in these patients are shown in **Supplementary Tables S1** and **S2**, respectively. Following 1:1:1 PSM, the baseline characteristics of the three groups are shown in **Table 2**. After matching, there were no statistically significant differences between the groups in terms of sex, age, transmission route, WHO clinical stage, baseline VL, baseline CD4^+^ T cell count, or initial ART regimen.

**Table 2:** Baseline characteristics of patients in three matched groups after PSM (n=195 per group).

Characteristic	PDR-negative group	V179D/E group	NNRTI-DR group	*P* value
Age, median (IQR)	40.0 (31.0-54.0)	37.0 (31.0-50.0)	39.0 (33.0-52.0)	0.376
Sex				0.896
Male	169 (86.7%)	172 (88.2%)	171 (87.7%)	
Female	26 (13.3%)	23 (11.8%)	24 (12.3%)	
Route of infection				0.612
same-sex sexual behavior	106 (54.4%)	120 (61.5%)	116 (59.5%)	
Heterosexual intercourse	74 (37.9%)	64 (32.8%)	64 (32.8%)	
Others	15 (7.7%)	11 (5.6%)	15 (7.7%)	
WHO clinical stage				0.927
I and II	19 (9.7%)	19 (9.7%)	21 (10.8%)	
III and IV	176 (90.3%)	176 (90.3%)	174 (89.2%)	
CD4^+^ T cell count before ART (cells/μL), median (IQR)	180.0 (65.0-318.0)	166.0 (50.0-317.0)	187.0 (29.0-342.0)	0.845
VL before ART (log10 copies/mL), median (IQR)^a^	5.2 (4.4-5.7)	5.3 (4.7-5.9)	5.3 (4.8-5.7)	0.219
ART regimen at baseline				0.530
2NRTIs+NNRTI	114 (58.5%)	101 (51.8%)	106 (54.4%)	
2NRTIs+PI	13 (6.7%)	21 (10.8%)	17 (8.7%)	
2NRTIs+INSTI	64 (32.8%)	64 (32.8%)	67 (34.4%)	
Simplified regimen	4 (2.1%)	9 (4.6%)	5 (2.6%)	
Subtype				<0.001
CRF01_AE	79 (40.5%)	38 (19.5%)	48 (24.6%)	
CRF07_BC	89 (45.6%)	25 (12.8%)	40 (20.5%)	
CRF55_01B	0 (0.0%)	108 (55.4%)	63 (32.3%)	
B	10 (5.1%)	5 (2.6%)	17 (8.7%)	
CRF08_BC	6 (3.1%)	7 (3.6%)	12 (6.2%)	
Others	11 (5.6%)	12 (6.2%)	15 (7.7%)	

IQR, interquartile range; PDR, pre-treatment drug resistance; WHO, World Health Organization; ART, antiretroviral therapy; VL, viral load; NRTI, nucleoside reverse transcriptase inhibitor; NNRTI, non-nucleoside reverse transcriptase inhibitor; PI, protease inhibitor; INSTI, integrase strand transfer inhibitor; CRF, circulating recombinant form.

a The PDR-negative, V179D/E, and NNRTI-DR groups include 68, 86, and 83 individuals, respectively.

**Figure 1:**
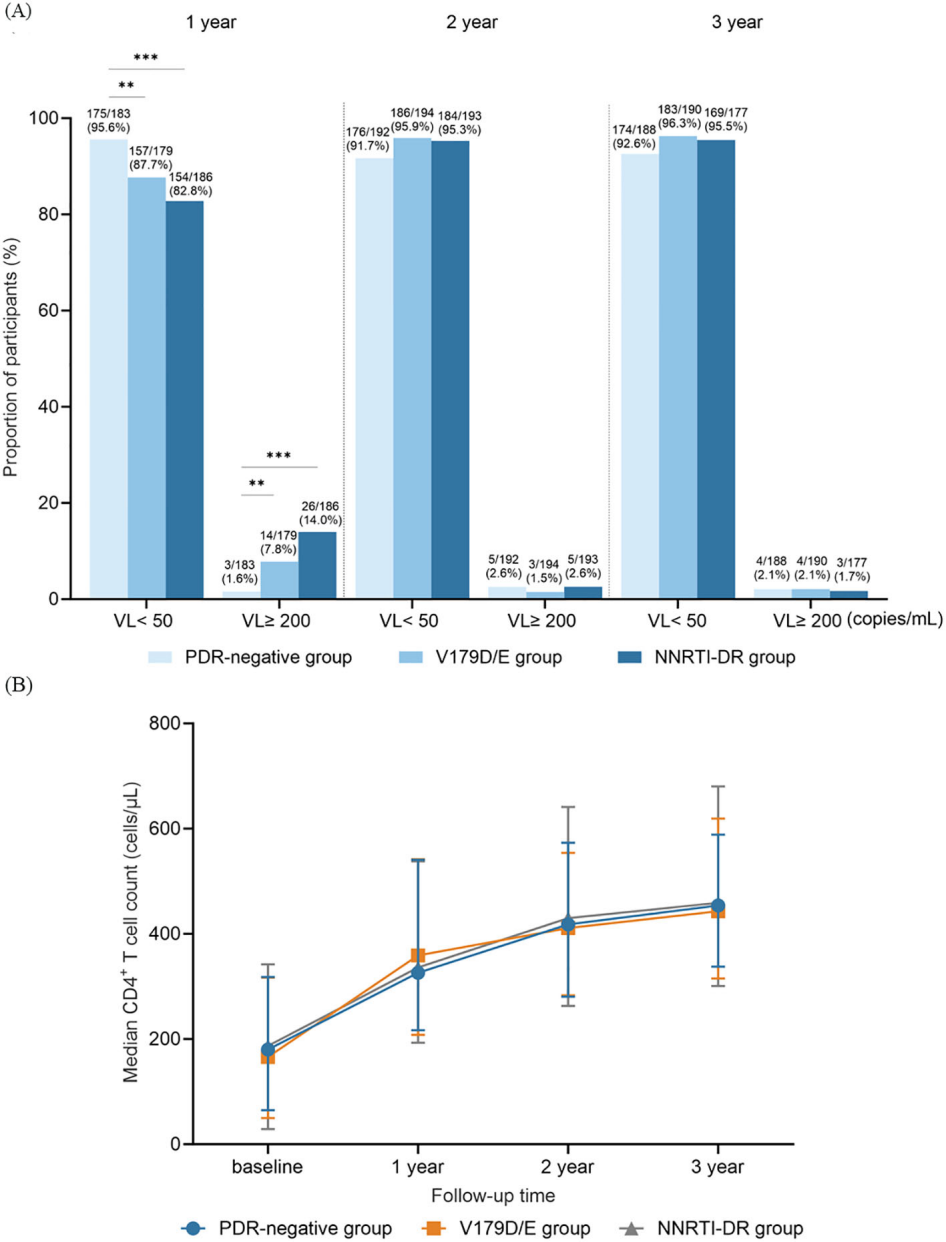
Longitudinal virological and immunological comparisons among three groups over a 3-year follow-up period. **(A)** Proportions of VS and VF patients in three group at years 1, 2, and 3. **(B)** Median CD4^+^ T cell counts in three group at years 1, 2, and 3. PDR, pre-treatment drug resistance; NNRT, non-nucleoside reverse transcriptase inhibitor; VL, viral load; VS, virological suppression; VF, virological failure. ** represents P < 0.01, and *** represents P < 0.001.

In the first year, the VS rate in the V179D/E group was significantly lower than that in the PDR-negative group (87.7% vs. 95.6%, *P* = 0.007) but higher than that in the NNRTI-DR group (87.7% vs. 82.8%, *P* = 0.238), although the latter difference was not statistically significant. Regarding VF, the V179D/E group had a higher rate than the PDR-negative group (7.8% vs. 1.6%, *P* = 0.006) but a lower rate than the NNRTI-DR group (7.8% vs. 14.0%, *P* = 0.066), with no statistically significant difference. These differences largely disappeared in the second and third years of follow-up (**Figure 1A**). In addition, CD4^+^ T cell count trajectories over the follow-up period were similar across the three groups, with no statistically significant differences (**Figure 1B**). 

The virological outcomes were comparable between the V179D and V179E groups (**Supplementary Figure S1A**), with a slightly higher suppression rate in the V179E group; however, the difference was not statistically significant. Regarding immunologic outcomes (**Supplementary Figure S1B**), the baseline CD4^+^ T cell count was significantly higher in the V179E group than in the V179D group (221.0 [IQR: 100.3–338.8] vs. 146.0 [IQR: 34.0–309.3], *P <*0.001). However, this difference gradually diminished during the follow-up, with no statistically significant differences observed at subsequent time points.

### 3.3 Analysis of risk factors for VF in the V179D/E group

A total of 73 individuals (73/626, 11.7%) in the V179D/E group experienced VF during follow-up. A comparison of the clinical characteristics of patients with and without VF is presented in **Supplementary Table S3**. Multivariate logistic regression analysis identified the use of 2NRTIs+NNRTI as a risk factor for VF (aOR = 1.91, *P* = 0.020). The specific NNRTI-based ART regimens used by patients are provided in **Supplementary Table S4**. Compared to a baseline CD4^+^ T cell count of <200 cells/μL, baseline CD4^+^ T cell counts of 200–350 cells/μL (aOR = 0.29, *P* < 0.001) and >350 cells/μL (aOR = 0.39, *P* = 0.010) were both significantly associated with a reduced risk of VF. For more detailed information, please refer to **Table 3**.

**Table 3:** Factors associated with VF in the V179D/E group.

Characteristic	Uni-variable analysis		Multi-variable analysis	
	OR (95% CI)	*P* value	aOR (95% CI)	*P* value
Age				
<30	Ref	Ref		
30-50	1.05 (0.53-2.09)	0.893		
>50	1.22 (0.58-2.57)	0.593		
Sex				
Male	Ref	Ref		
Female	1.37 (0.55-3.34)	0.498		
Route of infection				
same-sex sexual behavior	Ref	Ref		
Heterosexual intercourse	1.31 (0.78-2.21)	0.306		
Others	0.59 (0.17-1.98)	0.390		
WHO clinical stage				
I and II	Ref	Ref		
IV and V	1.04 (0.46-2.38)	0.924		
ART regimen at baseline				
2NRTIs+NNRTI	1.19 (0.71-2.00)	0.503		
Others^a^	Ref	Ref		
ART regimen at VF				
2NRTIs+NNRTI	**1.67 (0.98-2.86)**	**0.059**	**1.91 (1.11-3.29)**	**0.020**
Others^a^	Ref	Ref	Ref	Ref
CD4 count before ART (cells/μL)				
<200	Ref	Ref	Ref	Ref
200-350	**0.31 (0.16-0.61)**	**<0.001**	**0.29 (0.15-0.56)**	**<0.001**
>350	**0.41 (0.20-0.83)**	**0.014**	**0.39 (0.19-0.80)**	**0.010**
Type of DRMs				
V179D	Ref	Ref		
V179E	0.82 (0.48-1.39)	0.452		
HIV-1 subtype				
CRF01_AE	Ref	Ref		
CRF07_BC	0.64 (0.26-1.60)	0.340		
CRF55_01B	0.67 (0.35-1.26)	0.212		
Others	1.75 (0.72-4.26)	0.221		

VF, virological failure; OR, odds ratio; CI: confidence interval; WHO, World Health Organization; NRTI, nucleoside reverse transcriptase inhibitor; NNRTI, non-nucleoside reverse transcriptase inhibitor; ART, antiretroviral therapy; DRM, drug resistance mutation; CRF, circulating recombinant form.

a ART regimens classified as “others” include 2NRTIs+PI, 2NRTIs+INSTI, and simplified regimens. Variables with P < 0.1 in the univariable analysis were included in the multivariable model; bold values indicate statistically significant associations.

### 3.4 ADR profiles in the V179D/E group

**Figure 2:**
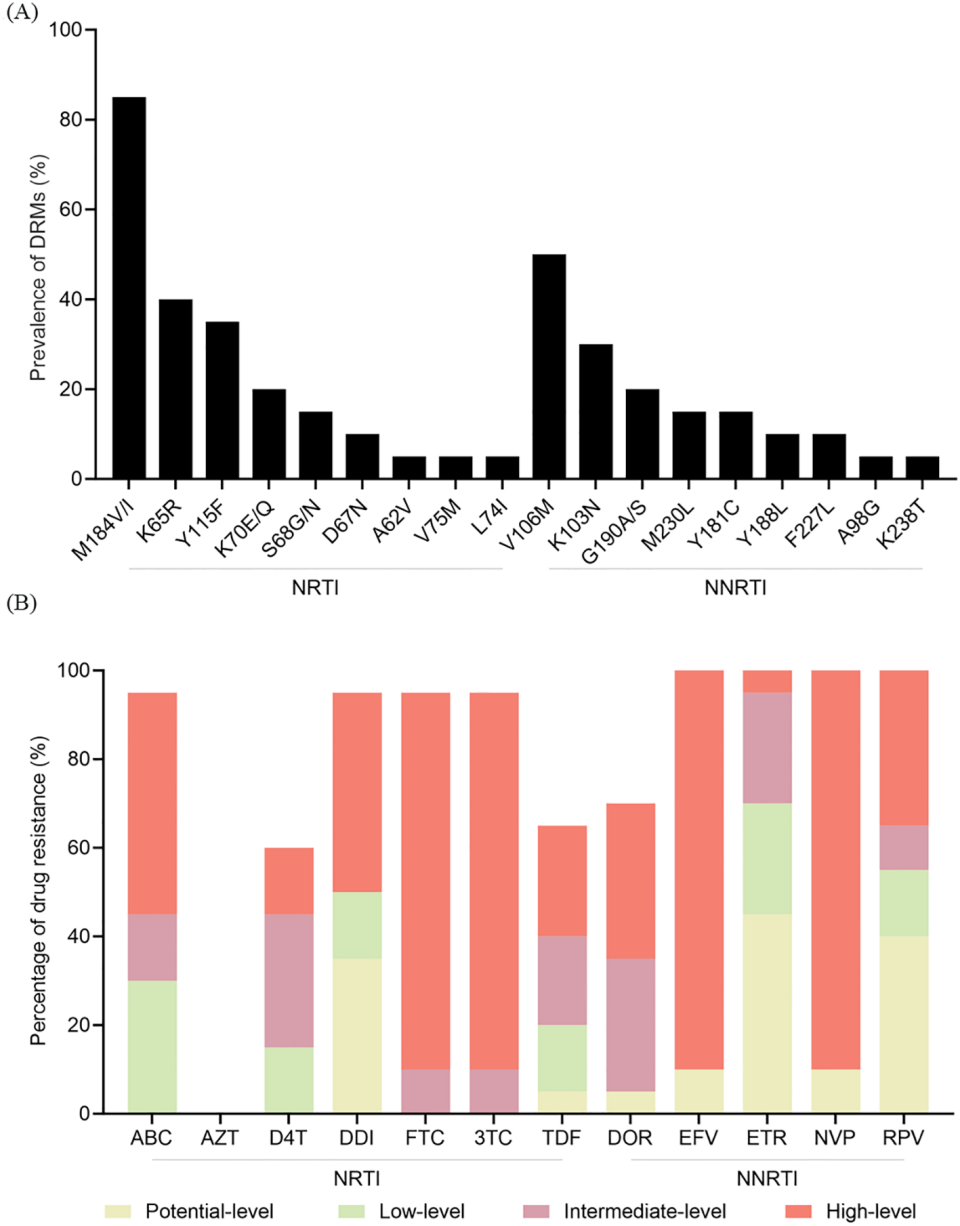
ADR profiles in the V179D/E group. **(A)** Newly emerged mutations identified during follow-up. **(B)** Cumulative drug resistance to various ARVs. NRTI, nucleoside reverse transcriptase inhibitor; NNRTI, non-nucleoside reverse transcriptase inhibitor; DRM, drug resistance mutation; ABC, abacavir; AZT, zidovudine; D4T, stavudine; DDI, didanosine; FTC, emtricitabine; 3TC, lamivudine; TDF, tenofovir disoproxil fumarate; DOR, doravirine; EFV, efavirenz; ETR, etravirine; NVP, nevirapine; RPV, rilpivirine; ADR, antiretroviral therapy; ARV, antiretroviral.

Of the 73 patients in the V179D/E group who experienced VF, 55 underwent ADR testing. Of these, 18 failed to be amplified, whereas the remaining 37 were successfully amplified. In addition to V179D/E, other new DRMs were identified in 20 patients (**Figure 2A**). These mutations were associated with resistance to both NRTIs and NNRTIs. Among the NRTI resistance mutations, M184V/I was the most common (85.0%), followed by K65R (40.0%). Among the NNRTI resistance mutations, V106M was the most frequently detected (50.0%), followed by K103N (30.0%). **Figure 2B** shows the levels of resistance to the various ARVs. All patients showed intermediate- to high-level resistance to emtricitabine (FTC) and lamivudine (3TC), 60% exhibited low-level resistance to tenofovir disoproxil fumarate (TDF), and 90% had high-level resistance to EFV and NVP. Notably, 85.0% (17/20) of the patients who developed new resistance mutations received a baseline ART regimen of 2NRTIs+NNRTI (3TC+TDF+EFV).

## 4 Discussion

In recent years, the prevalence of PDR has steadily increased, particularly in low- and middle-income countries (Inzaule et al., 2019). Studies have indicated that this trend is primarily driven by NNRTI-associated mutations (Su et al., 2025), among which V179D/E is the most frequently observed (Li et al., 2016; Zhang et al., 2021). Our previous study yielded similar results (Lv et al., 2024). In this study, the overall prevalence of V179D/E was 14.8%, which is similar to that reported in Shanghai, China (Zhang et al., 2024). V179E (10.5%) was much more common than V179D (4.3%), and 85.4% of V179E cases were linked to the CRF55_01B subtype, supporting earlier findings that V179E mainly circulates within this lineage (Liu et al., 2020; Lan et al., 2020).

V179D/E is polymorphic NNRTI-associated mutation that typically confer potential low levels of resistance. However, some studies have reported that patients with this mutation show poorer virological and immunological responses to EFV-based regimens than to PI- or INSTI-based therapies (Wang et al., 2023). Conversely, other studies have suggested that although V179D/E reduces NNRTI susceptibility *in vitro* (Vink et al., 2016), it does not significantly affect the clinical efficacy of NNRTI-based regimens or CD4^+^ T cell count recovery. Thus, the clinical significance of the V179D/E mutation remains controversial. Further studies are required to clarify their effects on first-line treatment efficacy, especially NNRTI-based regimens, which are crucial for optimizing therapy in ART-naïve individuals.

To assess the effect of V179D/E on treatment outcomes, two control groups were included: patients without PDR mutations (PDR-negative group) and patients with low or high NNRTI resistance (NNRTI-DR group). To minimize baseline differences that could influence outcomes, PSM was applied to control for sex, age, baseline CD4^+^ T-cell count, and initial ART regimen. In contrast, baseline VL was excluded because of missing data. In the first year of ART, the V179D/E group showed significantly lower virological efficacy than the PDR-negative group. Although the outcomes were slightly better than those of the NNRTI-DR group, the difference was not significant. No significant differences were observed between the three groups during the second and third years. These findings suggested that V179D/E may impair early virological responses. However, unlike previous studies (Wang et al., 2023), no significant differences in CD4^+^ T cell count recovery were observed, indicating a limited effect on immune reconstitution.

We further analyzed the risk factors for VF in the V179D/E group and found that the use of NNRTI-based regimens significantly increased the risk of VF. This finding is consistent with previous studies (Wang et al., 2023), indicating that although V179D/E confers only potential low-level resistance, it may still reduce the treatment efficacy of NNRTIs. Additionally, our results indicated that a baseline CD4^+^ T cell count above 200 cells/µL was associated with a lower risk of VF. These results underscore the need for caution when using NNRTI-based regimens in patients with V179D/E, especially those with CD4^+^ T cell counts below 200 cells/µL.

We evaluated ADR in patients from the V179D/E group who experienced VF and found that 54.1% (20/37) developed new DRMs. The most common NRTI-associated mutation was M184V/I, which causes high-level resistance to FTC and 3TC, followed by K65R, which confers intermediate-level resistance to TDF and abacavir (ABC). For NNRTIs, V106M was the most frequent, followed by K103N, both of which are linked to high-level resistance to EFV and NVP. Notably, seventeen of the 20 patients who developed new DRMs were on NNRTI-based regimens. All 17 of these patients were treated with the regimen 3TC+TDF+EFV, which likely explains why these specific mutations occurred. This suggests that continuing NNRTI therapy in the presence of V179D/E may not only reduce the initial virological response but also increase the risk of further resistance, complicating future treatment.

This study had some limitations. First, as this was a single-center retrospective study, the findings may not be widely applicable and require confirmation in larger multicenter studies. Second, drug resistance testing in this study was conducted using Sanger sequencing, which has a detection threshold of approximately 15%–20%, limiting the ability to detect low-frequency variants (Capina et al., 2020). As these mutations may still contribute to VF (Stella-Ascariz et al., 2017; Milne et al., 2019), their potential impact on treatment outcomes cannot be excluded. Additionally, as 99.5% of patients in the V179D/E group were treated with an EFV-based regimen, the conclusions may be more applicable to this treatment regimen, and the effect of other regimens could not be adequately assessed due to the small number of patients on other regimens.

## 5 Conclusions

Our findings indicated that the V179D/E mutation has a relatively high prevalence in ART-naïve patients with HIV-1 in Guangzhou, China. This may reduce the early virological response to NNRTI-based regimens, especially in patients treated with an EFV-based regimen, and increase the risk of acquired resistance mutations, especially in patients with low baseline CD4^+^ T cell counts.

## Data Availability

The original contributions presented in the study are included in the article/**Supplementary Material**, further inquiries can be directed to the corresponding author/s.

## Supplementary material

Supplementary Figure S1 Virological and immunological comparisons among V179D and V179E groups over a 3-year follow-up period. **(A)** Proportions of VS and VF patients in the two groups at years 1, 2, and 3. **(B)** Median CD4+ T cell counts in the two groups at years 1, 2, and 3. VL, viral load; VS, virological suppression; VF, virological failure. * represents P < 0.05.
