# Supplementary figures and images for: Impact of V179D/E mutations on antiretroviral therapy outcomes in people living with HIV-1: a 3-year retrospective study
Source: Front Cell Infect Microbiol. 2025 Dec 4;15:1691715. doi: 10.3389/fcimb.2025.1691715 (PMC12711782; doi:10.3389/fcimb.2025.1691715)

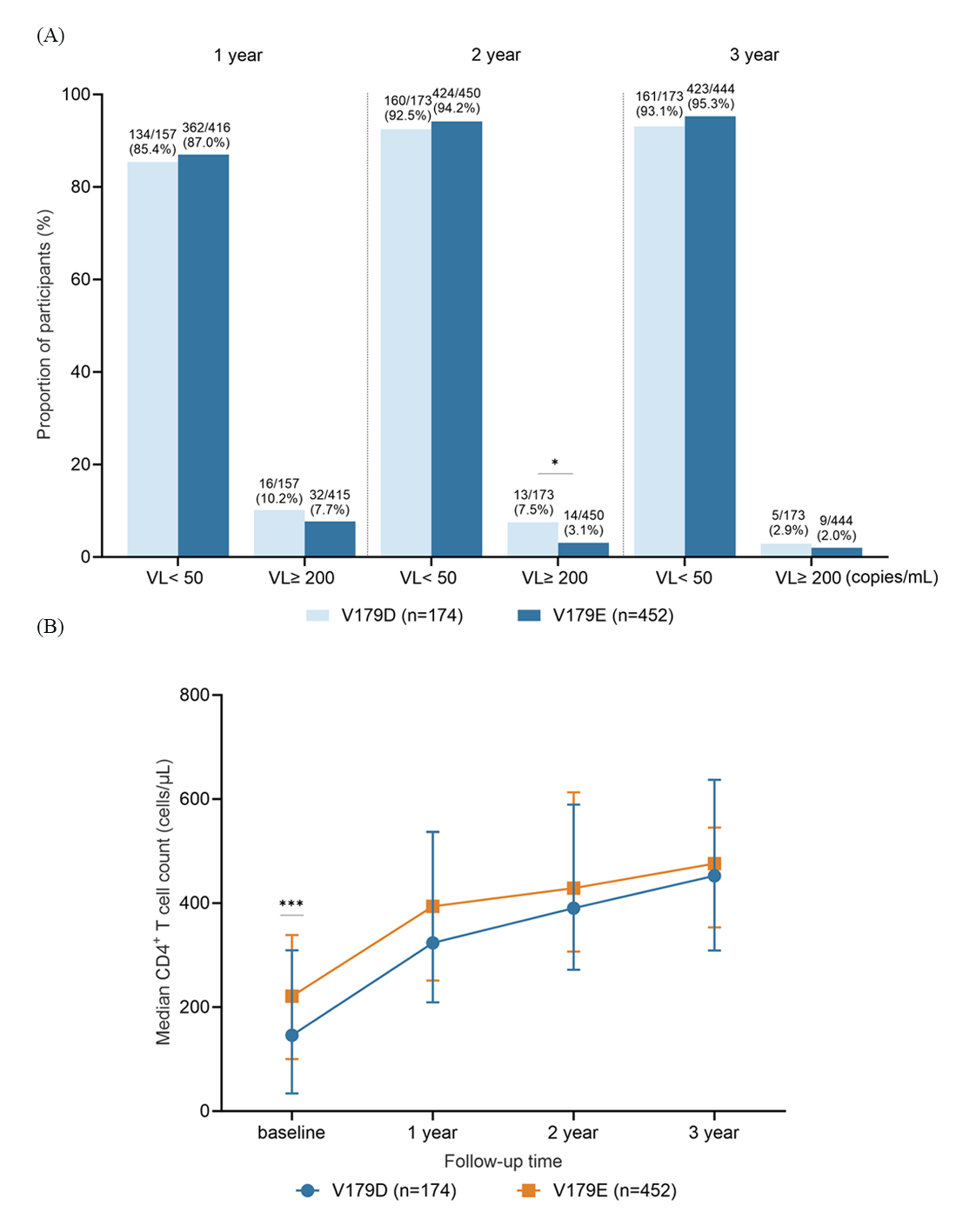

Supplement: Supplementary Figure S1 — Virological and immunological comparisons among V179D and V179E groups over a 3-year follow-up period. (A) Proportions of VS and VF patients in the two groups at years 1, 2, and 3. (B) Median CD4+ T cell counts in the two groups at years 1, 2, and 3. VL, viral load; VS, virological suppression; VF, virological failure. * represents P < 0.05. [file Image1.tif]
