# Supplementary material for: Impact of V179D/E mutations on antiretroviral therapy outcomes in people living with HIV-1: a 3-year retrospective study
Source: Front Cell Infect Microbiol. 2025 Dec 4;15:1691715. doi: 10.3389/fcimb.2025.1691715 (PMC12711782; doi:10.3389/fcimb.2025.1691715)
Supplement: Supplementary file 2 [file Table1.docx]

**FIGURE S1** virological and immunological comparisons among V179D and V179E group over a 3-year follow-up period. (a) Proportions of VS and VF patients in two groups at years 1, 2, and 3. (b) Median CD4^+^ T cell counts in two groups at years 1, 2, and 3. Abbreviations: VL, viral load; VS, virological suppression; VF, virological failure.

**TABLE S1** NNRTI-associated mutations in patients with NNRTI DR (n=195)

| NNRTI-associated mutations | Number | Percentage (%) |
| --- | --- | --- |
| E138G/A/K | 100 | 51.3 |
| K103N/Q | 36 | 18.5 |
| V106I/M/A | 25 | 12.8 |
| V179D/E/L | 81 | 41.5 |
| G190A/S | 7 | 3.6 |
| Y181C/L | 7 | 3.6 |
| A98G | 6 | 3.1 |
| K101E | 5 | 2.6 |
| V108I | 4 | 2.1 |
| Y188L/C | 2 | 1.0 |
| H221Y | 1 | 0.5 |
| K238T | 1 | 0.5 |
| M230L | 1 | 0.5 |

Abbreviations: NNRT, non-nucleoside reverse transcriptase inhibitor; DR, drug resistance.

**TABLE S2** Different resistance levels to NNRTIs in the NNRTI DR group (n=195)

| NNRTIs | S, n (%) | P, n (%) | L, n (%) | I, n (%) | H, n (%) |
| --- | --- | --- | --- | --- | --- |
| DOR | 144 (73.8) | 12 (6.2) | 30 (15.4) | 5 (2.6) | 4 (2.1) |
| EFV | 25 (12.8) | 33 (16.9) | 72 (36.9) | 23 (11.8) | 42 (21.5) |
| ETR | 37 (19.0) | 75 (38.5) | 75 (38.5) | 8 (4.1) | 0 (0.0) |
| NVP | 13 (6.7) | 37 (19.0) | 69 (35.4) | 19 (9.7) | 57 (29.2) |
| RPV | 37 (19.0) | 19 (9.7) | 117 (60.0) | 18 (9.2) | 4 (2.1) |

Abbreviations: NNRT, non-nucleoside reverse transcriptase inhibitor; DR, drug resistance; S, susceptible; P, potential low-level; L, low-level; I, intermediate-level; H, high-level; DOR, doravirine; EFV, efavirenz; ETR, etravirine; NVP, nevirapine; RPV, rilpivirine.

**TABLE S3** Clinical characteristics of patients with and without VF in the V179D/E group

| Characteristic | Patients with VF（n=73） | Patients without VF（n=553） | *P* value |
| --- | --- | --- | --- |
| Age, median (IQR) | 40.0 (32.5-56.0) | 40.0 (31.0-52.0) | 0.298 |
| <30 | 12 (16.4%) | 99 (17.9%) | 0.818 |
| 30-50 | 38 (52.1%) | 299 (54.1%) |  |
| >50 | 23 (31.5%) | 155 (28.0%) |  |
| Sex |  |  | 0.450 |
| Male | 67 (91.8%) | 519 (93.9%) |  |
| Female | 6 (8.2%) | 34 (6.1%) |  |
| Route of infection |  |  | 0.340 |
| same-sex sexual behavior | 44 (60.3%) | 353 (63.8%) |  |
| Heterosexual intercourse | 26 (35.6%) | 159 (28.8%) |  |
| Others | 3 (4.1%) | 41 (7.4%) |  |
| WHO clinical stage |  |  | 1.000 |
| I and II | 7 (9.6%) | 55 (9.9%) |  |
| IV and V | 66 (90.4%) | 498 (90.1%) |  |
| ART regimen at baseline |  |  | 0.568 |
| 2NRTIs+NNRTI | 49 (67.1%) | 349 (63.1%) |  |
| 2NRTIs+PI | 4 (5.5%) | 47 (8.5%) |  |
| 2NRTIs+INSTI | 19 (26.0%) | 136 (24.6%) |  |
| Simplified regimen | 1 (1.4%) | 21 (3.8%) |  |
| ART regimen at VF |  |  | 0.168 |
| 2NRTIs+NNRTI | 52 (71.2%) | 330 (59.7%) |  |
| 2NRTIs+PI | 5 (6.8%) | 40 (7.2%) |  |
| 2NRTIs+INSTI | 13 (17.8%) | 121 (21.9%) |  |
| Simplified regimen | 3 (4.1%) | 62 (11.2%) |  |
| VL before ART (log10 copies/mL), median (IQR)^a^ | 5.7 (5.3-6.0) | 5.1 (4.6-5.7) | **<0.001** |
| CD4 count before ART (cells/μL), median (IQR) | 98.0 (28.5-253.5) | 218.0 (86.5-336.5) | **<0.001** |
| <200 | 51 (69.9%) | 248 (44.8%) | **<0.001** |
| 200-350 | 12 (16.4%) | 186 (33.6%) |  |
| >350 | 10 (13.7%) | 119 (21.5%) |  |
| Type of DRMs |  |  | 0.487 |
| V179D | 23 (31.5%) | 151 (27.3%) |  |
| V179E | 50 (68.5%) | 402 (72.7%) |  |
| HIV-1 subtype |  |  | 0.065 |
| CRF01_AE | 15 (20.5%) | 89 (16.1%) |  |
| CRF07_BC | 8 (11.0%) | 74 (13.4%) |  |
| CRF55_01B | 40 (54.8%) | 356 (64.4%) |  |
| Others | 10 (13.7%) | 34 (6.1%) |  |

Abbreviations: IQR, interquartile range; VF, virological failure; WHO, World Health Organization; NRTI, nucleoside reverse transcriptase inhibitor; NNRTI, non-nucleoside reverse transcriptase inhibitor; PI, protease inhibitor; INSTI, integrase strand transfer inhibitor; ART, antiretroviral therapy; DRM, drug resistance mutation; CRF, circulating recombinant form.

^a^The patients with and without VF include 29 and 233 individuals, respectively.

TABLE S4 Detailed NNRTI-based ART regimens in the V179D/E group

|  | Patients with VF | Patients without VF |
| --- | --- | --- |
| NNRTI-based ART regimen at baseline | n=49 | n=349 |
| 3TC+TDF+EFV | 46 (93.9%) | 338 (96.8%) |
| 3TC+ABC+EFV | 2 (4.1%) | 1 (0.3%) |
| 3TC/AZT+EFV | 1 (2.0%) | 8 (2.3%) |
| 3TC+TDF+NVP | 0 (0.0%) | 2 (0.6%) |
| NNRTI-based ART regimen at VF | n=52 | n=330 |
| 3TC+TDF+EFV | 50 (96.2%) | 315 (95.5%) |
| 3TC+ABC+EFV | 1 (1.9%) | 1 (0.3%) |
| 3TC/AZT+EFV | 1 (1.9%) | 6 (1.8%) |
| 3TC+TDF+ANV | 0 (0.0%) | 7 (2.1%) |
| 3TC+TDF+NVP | 0 (0.0%) | 1 (0.3%) |

Abbreviations: NNRTI, non-nucleoside reverse transcriptase inhibitor; ART, antiretroviral therapy; VF, virological failure; 3TC, lamivudine; TDF, tenofovir disoproxil fumarate; EFV, efavirenz; ABC, abacavir; AZT, zidovudine; NVP, nevirapine; ANV, ainuovirine.
